# Coat color affects the resilience against heat stress impacts on testicular hemodynamics, reproductive hormones, and semen quality in Baladi goats

**DOI:** 10.1186/s12917-023-03653-w

**Published:** 2023-08-04

**Authors:** Hossam R. El-Sherbiny, Nesrein M. Hashem, Elshymaa A. Abdelnaby

**Affiliations:** 1 Theriogenology Department, Faculty of Veterinary Medicine, Cairo University, Giza, 12211 Egypt; 2 Department of Animal and Fish Production, Faculty of Agriculture (El-Shatby), Alexandria University, Alexandria, 21545 Egypt

**Keywords:** Bucks, Climatic changes, Nitric oxide, Oxidative stress, Luteinizing hormone

## Abstract

Drastic climatic changes threaten animal productivity and prolificacy, whose adaptability is governed by its pheno- and genotypic traits. This study was aimed at investigating the effect of coat color on the adaptability of goat bucks under heat stress conditions from the perspectives of testicular blood flow (TBF) and biometry, reproductive hormones, and semen quality. Twenty bucks (*Capra hircus*) bearing different coat colors were selected from a large flock and divided into four equal groups (n = 5 each) as follows: black coat (BC; 100% black), brown coat (BrC; 100% dark brown), white coat (WC; 100% white), white-black coat (WBC; 50–60% white). Bucks were examined for TBF [Doppler ultrasonography and serum nitric oxide (NO)], testosterone (T) and luteinizing hormone (LH), seminal plasma oxidative biomarkers [catalase (CAT), total antioxidant capacity (TAC), and malondialdehyde (MDA)], and sperm traits percentages [progressive motility (PM), viability (SV), normal morphology (NM), and sperm concentration (SC) once a week for seven consecutive weeks (W1-W7) in the summer season (temperature humidity index = 88.4–92.2). Specifically, at W3-W7, darker bucks (BC and BrC) testicular volume, testicular colored area, T, NO, CAT, TAC, PM, SV, NM, and SC (W7 only) differed significantly (P < 0.05) by decrease than the lighter ones (WC and WBC). Both Doppler indices and serum MDA concentrations were elevated (*P* < 0.05) at W3-W7 in the BC and BrC bucks compared to WC and WBC groups. In conclusion, bucks with lighter coats were more resistant to the negative effects of HS on TBF, seminal oxidative biomarkers, and semen quality.

## Introduction

Climatic fluctuations are considered a mega-challenge for animal production and prolificacy, being the worst in heat stress (HS) circumstances [1]. Recently, there were witnesses of progressive elevations in ambient temperature covering not only the tropics and subtropics but also temperate and cold territories. Animal fertility is compromised during hot months, being the least when the temperature humidity index (THI) exceeds the thermotolerance capacity of their bodies [2]. HS impairs the testicular function with hemodynamics, semen quality, and fertilizing potential, mainly due to oxidative stress (OS) [3]. Huge efforts have been made to ameliorate HS’s negative impacts, including modifications in management, nutrition, and environment, as well as the pre-selection of heat-tolerant species or breeds for optimum productivity [4–6]. A body of evidence has indicated that heat tolerance is governed by pleiotropic factors including species, breed, altitude, season of birth, genetic variation, and coat type and color [7].

Coat color is governed by the melanocortin system under the effect of the proopiomelanocortin gene (POMC). POMC prohormone translation triggers melanocortin peptides [α-, β-, γ- melanocortin stimulating hormone (MSH), and adrenocorticotropic hormone (ACTH; [8]. Melanocortin receptors in the skin bind either with melanocortin peptides (forming black coat color eumelanin) or agouti signaling protein (melanocortin antagonist, forming yellow coat color pheomelanin) [9]. It has been reported that darker males are more sexually active than lighter ones, owing to the higher androgen levels in darker males’ circulation [10]. Recently, Nejad et al. [11] proved that white-colored cows were more stressed by cold weather than black ones as indicated by higher cortisol and lower serotonin levels in their hair samples. Contrary data indicated that white-colored cows presented more resilience against HS conditions than black cows [12, 13]. Hence, the selection of males with phenotypic traits that make them more tolerant to adverse environmental stressors is beyond crucial for overwhelming its undesirable impacts on testicular functions and male reproductive patterns.

Since bucks have a diversity of coat colors ranging from dark to white and mixed; the authors hypothesized that coat color would affect their reproductive competence under HS circumstances. The hypothesis was tested by monitoring the testicular hemodynamics and volume, circulating hormones and oxidative biomarkers, and semen traits throughout the whole spermatogenesis (47 days) in different colored bucks under environmental HS conditions (summer season).

## Materials and methods

### Bucks and management

Twenty Baladi bucks of 2.4 ± 0.3 years of age with an average body weight of 42.3 ± 2.3 kg were selected for the current investigation. Bucks were normal breeders, having fertile records and being free from internal and andrological problems. Bucks were housed indoors with free exit an hour before sunset for 30 min daily. They were fed a balanced diet as per the NRC (2007) requirements, composed of green fodder (1.25 kg/head/day) and pelleted concentrates (400 g/head/day) with ad libitum access to water and mineral licks. The percentage of the animals’ body weight fed to bucks (concentrates) = amount of concentrates/average body weight * 100 = 0.4/42.3*100 = 0.94%.

### Study design

The present investigation was performed during the summer season (July-August 2021). Bucks were selected and divided (n = 5 each) following their coat color into white (WC, 100% white), black (BC, 100% black), white and black (WBC, 60–70% white), and brown (BrC, 100% brown). The bucks’ heat stress level was assessed based on the temperature-humidity index (THI) as per the formula of THI = (1.8*T + 32) – {(0.55 − 0.0055*RH)(1.8*T − 26), where T is the ambient temperature (^o^C) and RH-relative humidity (%) [14]. Calculated THI values (outdoor shed) were between 88.4–92.2, which indicated heat stress circumstances [15]. The heat stress that examined bucks were exposed ranged from 6–9 h/day throughout the study timeline. Once/week (W1-W7), Bucks were examined for testicular hemodynamics, volume, hormonal, biochemical analysis, and semen quality. The study timeline (7 weeks) was for spermatogenesis in goats. Environmental parameters (temperature, relative humidity, temperature humidity index, wind speed, and solar radiation) in Giza city, Giza governorate, Egypt throughout the study timeline as obtained from the meteorological authority, Cairo, Egypt are presented in Table 1.

**Table 1:** Environmental parameters (temperature, relative humidity, temperature humidity index, wind speed, and solar radiation) at Giza city, Giza governorate, Egypt during the study timeline (W1-W7; July and August 2021)

Environmental parameters	Week 1	Week 2	Week 3	Week 4	Week 5	Week 6	Week 7
**Temperature (** ^**o**^ **C)**	38.00 ± 3.36	36.9 ± 0.89	38.04 ± 0.84	38.71 ± 1.31	40.11 ± 1.39	38.9 ± 0.89	38.4 ± 1.21
**Relative humidity (%)**	48.50 ± 0.35	55.51 ± 0.43	55.01 ± 0.51	56.75 ± 0.64	46.52 ± 0.49	59.5 ± 0.71	61.25 ± 0.91
**Temperature humidity index**	88.4 ± 1.32	88.5 ± 0.96	89.9 ± 1.23	91.2 ± 0.83	90.5 ± 0.75	92.2 ± 0.65	91.9 ± 0.37
**Solar radiation (W/m** ^**2**^ **)**	925 ± 65	895 ± 54	955 ± 63	973 ± 46	951 ± 36	1012 ± 117	987 ± 87
**Wind speed (m/s)**	3.32 ± 0.82	2.87 ± 0.49	3.45 ± 1.20	3.41 ± 0.88	3.01 ± 0.81	2.80 ± 0.72	3.07 ± 0.55

Data is presented as weekly means ± standard deviation. W/m^2^ = Watts/ square meter; m/s = meter/second. Temperature humidity index = (1.8*T + 32) – {(0.55 − 0.0055*RH) (1.8*T − 26), considered as T and RH for ambient temperature (^o^C) and relative humidity (%), respectively [14]

### Testicular ultrasonography

Color Doppler ultrasonography was accredited for testicular hemodynamic (TH) assessment [3]. To begin with, scrotal hair covering the testes and the spermatic cord was clipped and shaved. Controlling of the examined bucks was performed with a co-worker’s aid and without any sedatives. On action, the linear ultrasound probe (6-7.5 MHz; EXAGO, ECM co., France) was placed on the lateral surface of the testis (testicular length, cm; TL) and crossed 90 ^o^ (testicular width, cm; TW) followed by probe crossing on the anterior border of the testis (thickness, cm; TT). Testicular volume (TV. cm^3^) was calculated using the following formula: TV = 4/3π TL*TW*TT [16]. For TH evaluation, the device probe was obliquely placed on the attached pole of the testis, seeking visualization of the testicular artery vascular cone, followed by activation of color flow mode to assess the total-colored area (TCA) within the testicular vascular cone in the spermatic cord area. Adobe Photoshop 64 CC software was applied to estimate the coloration in the frozen images that was later presented as pixels. Pulsed-wave Doppler was performed for measurement of pulsatility and resistive indices (PI and RI) of blood flow within the testicular artery. A minimum of three consecutive waves per testis is required for Doppler indices measurement [16].

### Serum harvesting and hormonal analysis

Serum samples were obtained by centrifugation of the plain-tubed jugular blood (5 ml) at 3000 RPM for 15 min, followed by deep-freezing at -20 ^o^C until further analysis. Testosterone (T) and LH levels were measured following radioimmunoassay utilizing commercial goat-specific ELISA kits (SunLong Biotech Co., China) with an intra-assay variance factor of 10–12% and minimal detectable levels of 0.05 ng/ml. All serum samples were analyzed after the study ended; therefore, there was no inter-assay variance factor [17].

### Semen quality assessment

Ejaculates were obtained using an electro-ejaculator in a 37 ^o^C prewarmed falcon tube (50 ml) once/week at 8:00 AM. Immediately, the ejaculate volume was measured by a micropipette. Sperm forward movement was assessed by placing a 2 µl of a diluted semen sample [1:20; sodium citrate dihydrate 2.9%, (v/v)] on a 37 ^o^C preheated glass slide and cover slipped, utilizing a heated-stage (37 ^o^C) phase-contrast microscopy (Olympus, Japan), the percentage of sperm rectilinear motility in a minimum of five fields [18]. Sperm motility was assessed on a 5-point scale, and the presented data were for the average of each group (n = 5). Sperm plasmalemma integrity was assessed using the eosin-nigrosin staining technique, where 1.67 g eosin was mixed with 10 g nigrosine in a 100 ml sodium citrate dihydrate (2.9%) solution. A mixed drop [1:3, v/v)] of the stain and diluted semen was spread on a prewarmed slide (37 ^o^C) and examined using a phase-contrast microscope (400x) for stain uptake (dead, pinkish sperm) or not (viable, colorless sperm). Morphological evaluation was performed using the slide of membrane integrity evaluation (1000x, oil immersion lens) for detection the of sperm abnormalities (head defects, midpiece defects, cytoplasmic droplets, and tail defects). A minimum of 300 sperm/slide were examined routinely in a duplicate smear for validity assurance. The data for membrane integrity and morphology were expressed as percents 0-100 [19].

### Assessment of seminal plasma oxidative biomarkers

Seminal plasma (SP) was harvested from the centrifuged neat semen samples (2000 g for 15 min at 4 ^o^C) and stored at -20 ^o^C for further assessment. For inter-assay variations exclusion, all the oxidative biomarkers (W1-W7) were assayed on the last day of the experiment. TAC (mM/L), MDA (mM/ml), and NO (µM/L) concentrations were measured calorimetrically whereas, CAT (U/L) activity was measured using commercial photometric kits following the producer’s instructions (Bio-diagnostics Co., Gizah, Egypt), specifically at a wavelength of 510, 534, 534, and 520 nm (Spectrophotometer, USA) [20, 21].

### Statistical analysis

Shapiro-Wilk and Levene tests were initially assigned for normality and homogeneity data assurance, respectively, which was accredited with a probability over 0.05. Repeated measures two-way ANOVA was used to examine the effect of coat color (fixed factor; WC, BC, BrC, and WBC) on testicular hemodynamics (RI, PI, and TCA) and volume, hormones (T and LH), semen quality (PM, SV, NM, and SC), NO and seminal oxidative biomarkers (CAT, TAC, and MDA) as dependent variables during the experimental timeline (time effect; W1-W7). Results with a probability less than 0.05 were considered significant. All statistical analyses were performed using the statistical package for the social sciences (SPSS 25, USA).

## Results

### Effect of coat color on testicular volume and hemodynamics

**Fig. 1:**
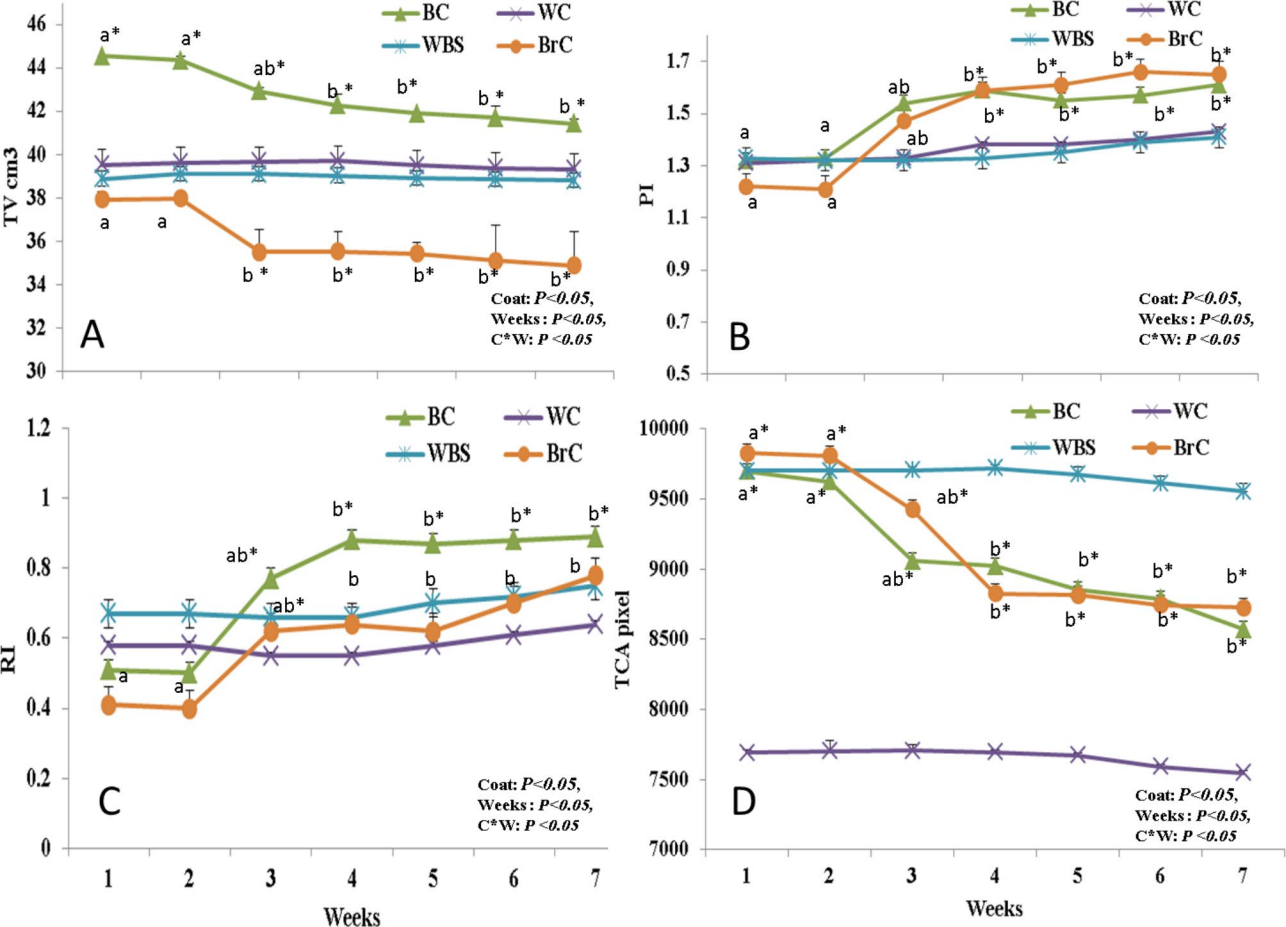
The changes in testicular volume (TV, cm, A), pulsatility index (PI, B), resistance index (RI, C), and the total-colored area (TCA; pixel, D) in bucks with different coats colors (WC, BC, WBC, and BrC; n = 5 each) in the summer season (W1-W7; THI > 88.3). Data are presented as means ± standard errors of the means (SEM). Wks = weeks, WC = white colored, BC = black colored, WBC = white-black colored (50–60% white), BrC = brown colored, THI = temperature humidity index. Means with different superscripts are significantly different at P < 0.05. *Values in each measure are different at least at P < 0.05 between the groups (BC and BrC Vs WC and WBS)

Testicular volume and hemodynamics (RI, PI, and TCA) were affected (*P* < 0.05) by coat color, weeks, and their interaction (Fig. 1). In detail, there were significant (*P* < 0.05) decreases in the TV values noted at W3 and have continued gradually to the end of the study at W7 in the BC and BrC bucks compared to their means at W1. Particularly at W3-W7, darker bucks’ (BC and BrC) TV means differed significantly (*P* < 0.05) by declination compared to the lighter ones (WC and WBC). Throughout the study timeline (W1-W7), there were no significant alterations in the TV records of the WC and WBC bucks (Fig. 1A).

Regarding the testicular hemodynamics alterations, the values of PI in the BC and BrC started to increase at W3, peaked (*P* < 0.05) at W4, and held on till the study ended (W7). The pattern of PI increases at W3-W7 in the BC and BrC bucks was more prominent (*P* < 0.05) compared to the WC and WBC groups. However, the RI increase was more pronounced in the BC than the BrC bucks that had the RI peak at W7. The RI and PI values of WC and WBC showed non-significant increases during the experimental time points (Fig. 1B C). Darker bucks witnessed a drastic drop in the TCA starting at W3 and being the least at W7. In the same manner, the TCA value decrease was prominent (*P* < 0.05) at W3-W7 in the BC and BrC groups compared to the WC and WBC bucks. Non-significant decreases were noted in the WC and WBC bucks throughout the study timeline (Fig. 1D).

### Effect of coat color on concentrations of T, LH, and NO

**Fig. 2:**
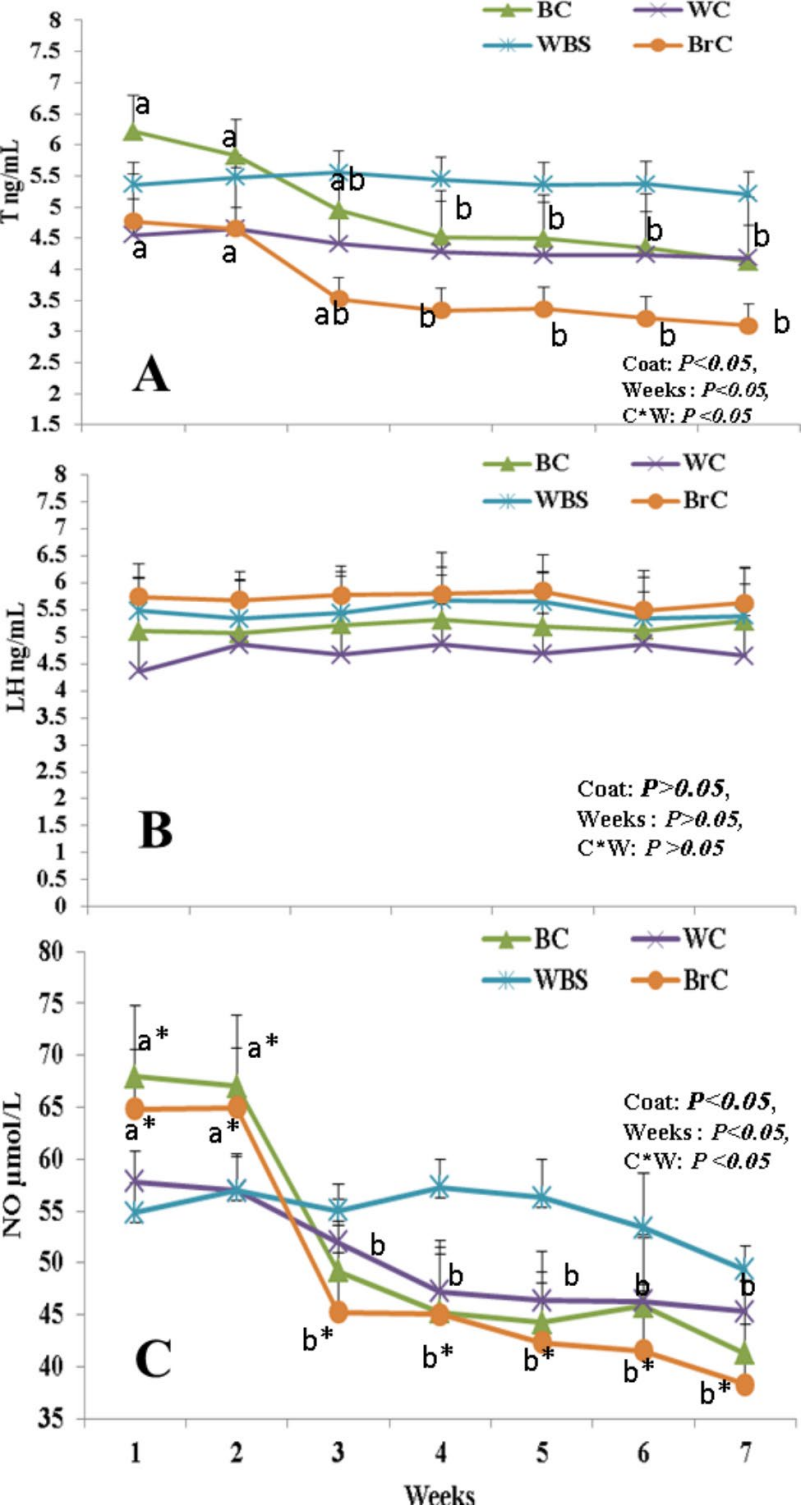
The changes in testosterone (T.ng/mL, A), luteinizing hormone (LH, ng/mL, B), and nitric oxide levels (NO,µmol/L, C) in bucks with different coat colors (WC, BC, WBC, and BrC; n = 5 each) in the summer season (W1-W7; THI > 88.3). Data are presented as means ± standard errors of the means (SEM). Wks = weeks, WC = white colored, BC = black colored, WBC = white-black colored (50–60% white), BrC = brown colored, THI = temperature humidity index. Means with different superscripts are significantly different at P < 0.05. *Values in each measure are different at least at P < 0.05 between the groups (BC and BrC Vs WC and WBS)

Concentrations of T (Fig. 2A) and NO (Fig. 2C) were affected by coat color, weeks, and coat color × week interaction (*P* < 0.05, for all), but not LH (Fig. 2B). Levels of T in the BC and BrC bucks gradually decreased reaching the significant point at W4 (*P* < 0.05) and became steady onward. Compared to the WC and WBC bucks, the T concentrations in the BC and BrC bucks declined clearly (*P* < 0.05) at W4-W7. There were no significant alterations in the means of T in the WC and WBC among the studied time points.

Earlier than the T changes, the NO concentrations were abruptly dropped at W3 (*P* < 0.05) and found their way to W7 in a constant attitude. In a descending manner, the pattern of NO decreases in all groups was as follows: BrC, BC, and WC, respectively. However, the significance (*P* < 0.05) of the NO decreasing pattern was observed between the darker ducks (BrC and BC) and the lighter ones (WC and WBC). Whereas the changes in the NO levels among the experimental time points in the WC and WBC were nonsignificant.

### Effect of coat color on seminal oxidative biomarkers (CAT, TAC, and MDA)

**Fig. 3:**
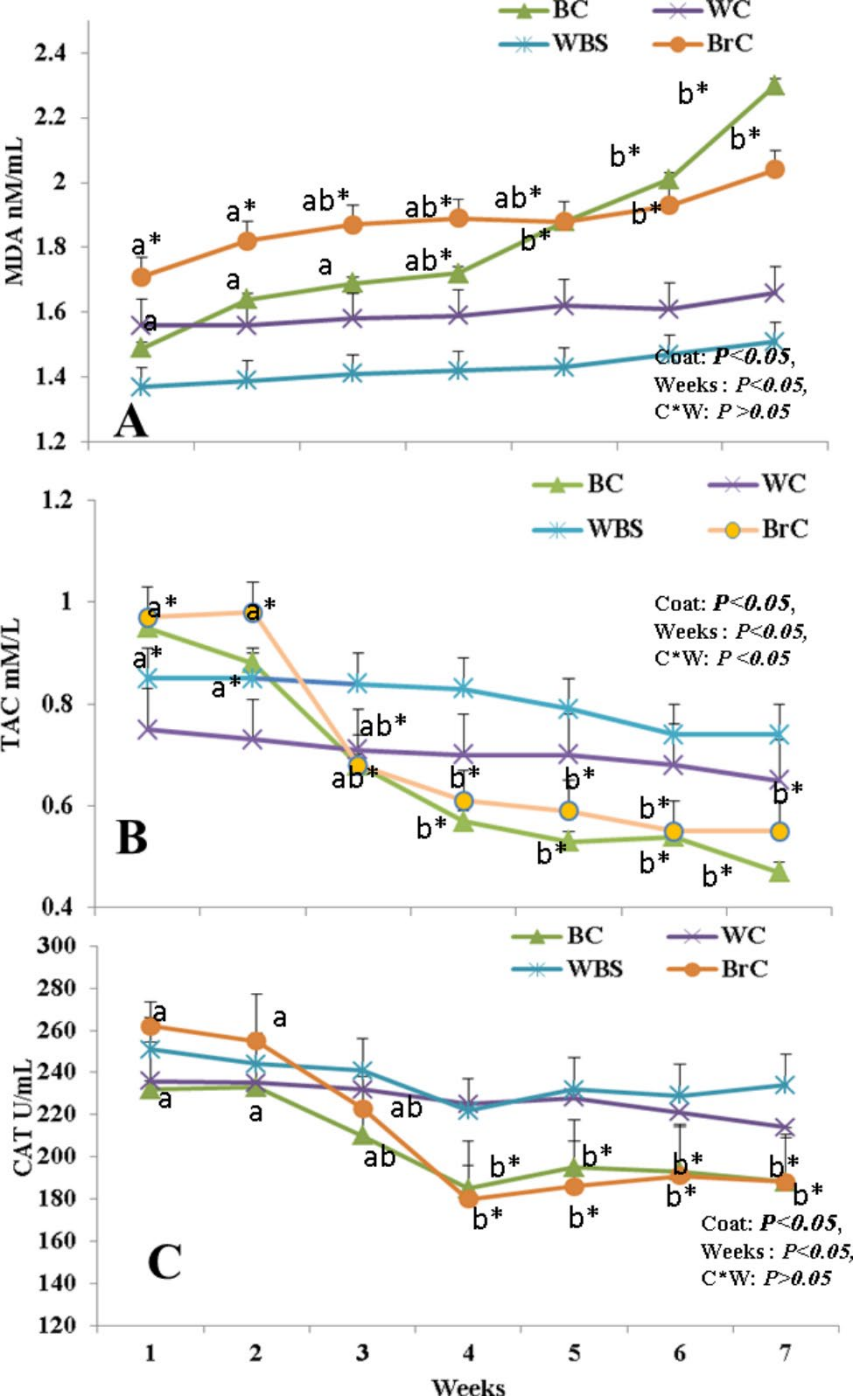
The changes in seminal plasma levels of malondialdehyde (MDA; nM/mL, A) and total antioxidant capacity (TAC; mM/L, B) and activities of catalase (CAT; U/Ml, C) in bucks with different coat colors (WC, BC, WBC, and BrC; n = 5 each) in the summer season (W1-W7; THI > 88.3). Data are presented as means ± standard errors of the means (SEM). Wks = weeks, WC = white colored, BC = black colored, WBC = white-black colored (50–60% white), BrC = brown colored, THI = temperature humidity index. Means with different superscripts are significantly different at P < 0.05. *Values in each measure are different at least at P < 0.05 between the groups (BC and BrC Vs WC and WBS)

There were effects of coat color and weeks (*P* < 0.05, for both) on the MDA concentrations in the bucks’ SP (BC, BrC, WC, and WBC) under HS conditions (Fig. 3). MDA (nM/mL) levels (Fig. 3A) of the BC and BrC bucks increased significantly (P < 0.05) at W6-W7 compared to their values at W1-W5; while in the WC and WBC, there were no significant changes among the study weeks. In addition, TAC (mM/L) concentrations (Fig. 3B) and CAT (U/Ml) activity (Fig. 3C) were affected by color, weeks, and their interaction (*P* < 0.05), showing the same trend by decreasing specifically at W4-W6 in the BC and BrC groups compared to the WC and WBC bucks, which were merely constant throughout the experiment.

### Effect of coat color on semen traits

As presented in Tables 2 and 3, color coat, weeks of HS conditions, and the interaction between them affected (*P* < 0.05, for all) sperm traits of the experimental bucks, including sperm progressive motility (PM, %), viability (SV, %), and normal morphology (NM, %), but not sperm concentration (SC, 10^9^ cell/ml), which was affected only by weeks. All bucks bearing different colors witnessed a decrease in PM % starting from W3 for BC and BrC bucks and onward, being the least at W7, while PM % decreased later in WC and WBC at W6-W7 and W7, respectively, compared to their values at W1 (*P* < 0.05). There was specific timing (W3-W7) unveiling the significant decrease in PM % in darker bucks (BC and BrC) over the lighter ones (WC and WBC). In addition, starting at W3, SV % decreased obviously (*P* < 0.05) in the BC and BrC bucks, reaching the least significant values at W7, whereas the first significant (*P* < 0.05) point of SV % in WC and WBC was at W7. Furthermore, starting at W3 and W4 for BC and BrC, respectively, and ending at W7, SV % decreased markedly (*P* < 0.05) compared with WC and WBC. For NM%, there were decreases (*P* < 0.05) at W3-W7 for BC and BrC bucks and W5-W7 for WC and WBC compared with their means at W1. Specifically, at W4-W7, darker bucks possessed lower values of NM % than lighter ones (*P* < 0.05). Irrespective of their coat colors, all experimental bucks had the least significant decrease in their SC at W7 (*P* < 0.05) compared to other time points.

**Table 2:** Semen parameters (progressive motility [PM, %], and viability [SV, %]) of bucks with different coat colors (WC, BC, WBC, and BrC; n = 5 each) during summer season (W1-W7; THI > 88.3). Data are presented as means ± standard errors of means (SEM)

Wks	PM%				SV %			
	BC	WC	WBS	BrC	BC	WC	WBS	BrC
W1	86.00 ± 0.22 ^a^	85.00 ± 2.08^a^	86.50 ± 1.25^a^	82.00 ± 1.11 ^a^	90.00 ± 1.11^a^	90.40 ± 1.11^a^	89.40 ± 1.17^a^	88.50 ± 1.51 ^a^
W2	81.40 ± 0.55 ^a^	82.10 ± 1.74 ^a^	85.88 ± 1.18^a^	80.40 ± 1.24 ^a^	89.88 ± 0.55^a^	89.20 ± 2.32^a^	89.80 ± 1.33^a^	88.88 ± 0.74 ^a^
W3	70.80 ± 1.52 ^b*^	78.80 ± 2.77 ^ab^	82.00 ± 1.15^a^	71.55 ± 1.10 ^b*^	83.32 ± 0.74^b^	87.20 ± 0.36^a^	86.20 ± 0.52^a^	78.55 ± 0.77 ^b*^
W4	71.88 ± 1.66 ^b*^	77.44 ± 1.67^ab^	82.40 ± 1.11^a^	69.20 ± 1.25 ^b*^	81.02 ± 2.31^b*^	86.82 ± 0.55^a^	88.20 ± 0.74^a^	77.20 ± 0.22 ^b*^
W5	72.12 ± 1.92^b*^	77.65 ± 1.87^ab^	80.75 ± 1.32^ab^	66.65 ± 1.36^bc*^	79.24 ± 1.11^b*^	84.05 ± 1.32^ab^	88.31 ± 0.35^a^	76.53 ± 0.65^bc*^
W6	70.56 ± 2.36^b*^	75.71 ± 1.10^b^	79.10 ± 1.12^ab^	66.92 ± 0.58^bc*^	76.21 ± 1.31^b*^	84.3 ± 1.22^ab^	86.21 ± 0.34^a^	72.25 ± 0.84^bc*^
W7	63.42 ± 1.22^c*^	73.12 ± 1.36^b^	75.23 ± 1.09^b^	62.32 ± 1.68^c*^	71.12 ± 0.88^c*^	81.01 ± 1.01^b^	81.32 ± 1.01^b^	69.53 ± 0.24^c*^

Wks = weeks, WC = white colored, BC = black colored, WBC = white-black colored (50–60% white), BrC = brown colored, THI = temperature humidity index. Means with different superscripts in each column are significantly different at P < 0.05. *Values in each measure are different at least at P < 0.05 between the groups (BC and BrC VS WC and WBS)

**Table 3:** Semen variables (normal morphology [NM, %] and sperm cell concentrations [SC, 10^9^/ml]) of bucks with different coat colors (WC, BC, WBC, and BrC; n = 5 each) during summer season (W1-W7; THI > 88.3). Data are presented as means ± standard errors of means (SEM)

Wks	NM %				SC 10^9^/ml			
	BC	WC	WBS	BrC	BC	WC	WBS	BrC
W1	93.80 ± 1.33^a^	92.80 ± 1.81^a^	92.00 ± 0.85^a^	89.50 ± 1.73^a^	2.15 ± 0.11^a^	1.93 ± 0.04^a^	1.99 ± 0.22^a^	2.03 ± 0.02^a^
W2	90.80 ± 0.77^a^	89.90 ± 1.57^a^	89.00 ± 0.22^a^	86.00 ± 1.55^a^	2.10 ± 0.02^a^	1.91 ± 0.05^a^	1.98 ± 0.52^a^	2.01 ± 0.02^a^
W3	85.00 ± 0.74^b^	88.00 ± 0.24^a^	87.22 ± 0.41^ab^	81.50 ± 0.88^b^	2.10 ± 0.01^a^	1.85 ± 0.01^ab^	2.01 ± 0.63^a^	1.88 ± 0.14^ab^
W4	79.33 ± 0.55 ^b*^	85.85 ± 0.65 ^ab^	87.26 ± 0.63^ab^	79.88 ± 1.33^b*^	2.03 ± 0.25^ab^	1.86 ± 0.22^ab^	1.97 ± 0.01^a^	1.85 ± 0.07^ab^
W5	79.21 ± 1.21^b*^	85.05 ± 0.35^b^	85.35 ± 0.56^b^	77.32 ± 0.35^bc*^	1.97 ± 0.36^ab^	1.88 ± 0.04^ab^	1.92 ± 0.07^ab^	1.85 ± 0.03^ab^
W6	77.42 ± 1.72^b*^	84.01 ± 0.36^b^	84.32 ± 0.35^b^	77.65 ± 0.52^bc*^	1.83 ± 0.45^ab^	1.83 ± 0.32^ab^	1.91 ± 0.32^ab^	1.84 ± 0.74^ab^
W7	71.11 ± 1.02^c*^	83.04 ± 0.44^b^	84.02 ± 0.52^b^	73.36 ± 0.65^c*^	1.82 ± 0.32^b^	1.83 ± 0.57^b^	1.82 ± 0.02^b^	1.74 ± 0.12^b^

Wks = weeks, WC = white colored, BC = black colored, WBC = white-black colored (50–60% white), BrC = brown colored, THI = temperature humidity index. Means with different superscripts in each column are significantly different at P < 0.05. *Values in each measure are different at least at P < 0.05 between the groups (BC and BrC VS WC and WBS)

## Discussion

Nowadays, drastic fluctuations in climatic changes attract scientific concerns to explore definite solutions for the alleviation of its health hazards [22]. The selection of heat-tolerant phenotypic traits is crucial in males for optimum reproductive performance during heat stress (HS) conditions [23]. The color of the coat, from a morphological perspective, is crucial to the developed adaptation and is regarded as a qualitative measure demonstrating the genetic adaptability of animals to hot circumstances. Compared to animals with darker coats, those with lighter coats reflect between 50 and 60 percent of direct sun radiation and absorb less heat [24]. The West African dwarf goats’ fine, short, and straight hair aids in their adaptation to hot, humid surroundings. The color of their coat controls the amount of radiant heat load that is absorbed from the environment and reflected from their body [25]. Goats are homeothermic animals that are capable of regulating their body temperature when exposed to high temperatures. The respiratory system and the skin surface both function to dissipate excess heat [26]. Goats produce less metabolic heat when exposed to solar radiation up to 800 W/ m^2,^ and they produce more at levels above this because increased respiratory rate and flow slow down growth, output, and reproduction. However, the ratios of oxygen consumed, and carbon dioxide produced are kept constant [27]. Goats respond to HS through several mechanisms including behavioral, physiological, hormonal and molecular modulations [28].

This study monitored the testicular hemodynamics, circulating hormones, semen quality, and oxidative biomarkers in bucks bearing different coat colors (WC, BC, WBC, and BrC) throughout the 7-wk of spermatogenic cycle in the summer season (THI > 88.3) for selection of the most heat-tolerant bucks according to their coat color. In this study, the testicular hemodynamics (RI, PI, and TC) were affected by coat color, with the elevation of RI and PI values and a decrease in TC starting from W3 and onward in the BC and BrC groups compared to the WC and WBC groups. The increase in RI and PI and a decrease in TC could be interpreted by a higher vascular resistance and lower vascular flow and consequently lower testicular perfusion [29]. These results indicated that the lighter coat color bucks (WC and WBC) were resistant to the adverse impacts of heat stress on testicular hemodynamics than the darker bucks. Testicular blood flow reduction observed in the current study is corroborated by previous studies conducted on goats [30], rams [31, 32], and buffalo bulls [33]; these studies concluded that either environmental or induced testicular HS decreased its blood perfusion. Explanation of the first 2-wk effect of HS exposure on the testicular blood flow pattern of the examined bucks may be due to the compensatory mechanism exerted by the endogenous antioxidant systems that overcame the post-HS oxidative stress cascade followed by exhaustion from W3 and onward [34]. The latter theory is corroborated by the elevated seminal TAC and CAT levels during the first 2-wks of the experiment followed by a gradual decrease being the least at the end of the study. The NO levels were matched with the hemodynamic pattern starting to decline at W3 following the exhaustion of the antioxidant system. It was reported that higher free radicals (especially superoxide anion) generation in stressful conditions compulsively attack NO forming the high prooxidant peroxynitrite molecule and ultimately lower the circulatory NO levels [17, 35]. The NO being an endothelial modulator and vasodilator element, its low availability initiates vasoconstriction, and testicular blood flow declines [36, 37]. The privilage in lighter bucks over darker ones in heat stress tolerance may be due to the higher heat convection feature from one side [26], and genetic based heat adaptability from the other side [38]. Recently, Venkatesh et al. [38] explored a number of haplotypes among which the three (CCGG, TCGG, and CCTC) that are linked with white coat goats are associated with lower heat stress response (cortisol levels, rectal and skin temperature, and respiratory rate).

Electronic assessment of the testicular volume provides actual testicular dimensions irrespective of the adjacent scrotal and epididymal tissues [39]. At W4-W7 of the experiment, there was a significant decline in the TV values in the BC followed by BrC groups compared to WC and WBC groups. TV values decrease upon chronic heat stress exposure primarily due to apoptosis [40]. Hedia et al. [41] carried out a monthly evaluation of TV in rams and found that the least TV measurements during the time of heat stress conditions (summer season). As reviewed by Shahat et al. [5], a 30% of the antiapoptotic genes (BCL2) are reduced in bulls and mice just after 24 h after heat exposure. It was reported that chronic HS exposure deteriorates germinal epithelium, Sertoli, and Leydig population in rats [42]. In addition, HS decreases seminiferous tubules’ diameter and increases interstitial tissue space [43]. Moreover, the lower testicular blood flow noted in the present study might have affected the testicular fluid volume [44]. These are the possible explanations for the differences in TV between the darker and lighter bucks, the former of which were more susceptible to heat-stress [45]. Furthermore, it was reported that testosterone levels are positively correlated to TV; therefore, the decrease in T concentrations noted in the present study supports the TV changes [46].

Semen traits of the lighter coat bucks (WC and WBC) showed more resilience against HS effects than the darker coat bucks (BC and BrC) regarding the percentages of progressive motility, viability, normal morphology, and concentration, being the best in the WBC group. It has been reported that white-coat cows are less responsive to the adverse effects of HS than black-coat cows evidenced by lower serum cortisol concentrations [12]. As reviewed by Shahat et al. [5], the negative effect of HS is governed by the extent and duration of heat exposure, and the deteriorating effects may appear as early as 14 days post-exposure. Herein, all the experimental bucks were affected to some extent at W3 and down streamed to the last week of the experiment. The semen quality decline in darker coat bucks was faster and more intense than the lighter bucks, indicating a higher responsive pattern [47]. Lower seminal antioxidant potential (TAC, CAT) and higher lipid peroxide biomarker (MDA) in the darker coat bucks indicate an exhaustive effect of HS on the antioxidant defense.

The difference in response against HS in animals bearing different coat colors might be attributed to the percentage of solar and heat radiations absorbed by the skin, being the highest in darker animals [48]. However, in the present study, lighter bucks (WC and WBC) showed the best adaptability in prolonged HS circumstances compared to darker bucks. These results indicate that up to 40–50% black color mixed with white color did not affect the heat resilience of light coat goats. It has been reported that, in thermoneutral conditions, black males have higher sexual activity and androgen concentration than white ones [49]; however, black males are more vulnerable to HS due to the high absorption of heat by their coats [50]. Therefore, the higher adaptability of WBC bucks may be related to the higher natural reproductive performance of the black coat mixed with the higher HS resilience of the white coat. However, molecular studies are required to classify the bucks according to their color percentage (i.e., 100% white, 90%, 80%, 70% and so on) to define whether the heat-resilient genes are controlled by the color percent, or these genes have different expression pattern only with full color only (i.e., total black or total white). This study provided useful information in the selection of heat-tolerant phenotypic traits of the bucks aiming to alleviate the adverse effects of HS on the animals’ breeding program. As the number of animals in the current study was relatively low, it is necessary to ensure these outcomes on a large goat population.

## Conclusion

A buck’s coat color is associated with testosterone levels, sperm quality, and testicular blood flow and volume. The above indices were lower in darker bucks (BC and BrC) when compared to lighter-colored bucks (WC and WBC), especially at W3 during summer heat stress. Reductions in the examined testicular traits in the different groups could be ordered in a descending manner as follows: BC, BrC, WC, and WBC. Further studies are required to investigate the molecular bases affecting the adaptability against HS effects. In tropics and subtropics, it is recommended to integrate WC and WBC bucks under HS conditions into breeding system for optimum outcomes.

## Data Availability

The datasets used and/or analyzed during the current study are available from the corresponding author upon reasonable request.

## References

[1] Takahashi M (2012). Heat stress on reproductive function and fertility in mammals. Reprod Med Biol.

[2] Ahmad Para I, Ahmad Dar P, Ahmad Malla B, Punetha M, Rautela A, Maqbool I (2020). Impact of heat stress on the reproduction of farm animals and strategies to ameliorate it. Biol Rhyth Res.

[3] El-Sherbiny H, Abdelnaby E, El-Shahat K, Salem N, Ramadan E, Yehia S (2022). Coenzyme Q10 Supplementation enhances testicular volume and hemodynamics, reproductive hormones, sperm quality, and seminal antioxidant capacity in goat bucks under summer hot humid conditions. Vet Res Commun.

[4] Atta MS, Farrag FA, Almadaly EA, Ghoneim HA, Hafez AS, Al Jaouni SK (2020). Transcriptomic and biochemical effects of pycnogenol in ameliorating heat stress-related oxidative alterations in rats. J Therm Biol.

[5] Shahat A, Rizzoto G, Kastelic J (2020). Amelioration of heat stress-induced damage to testes and sperm quality. Theriogenology.

[6] Fadl AM, Abdelnaby EA, El-Sherbiny HR (2022). Supplemental dietary zinc sulphate and folic acid combination improves testicular volume and haemodynamics, testosterone levels and semen quality in rams under heat stress conditions. Reprod Domest Anim.

[7] Rashamol VP, Sejian V, Bagath M, Krishnan G, Archana PR, Bhatta R (2020). Physiological adaptability of livestock to heat stress: an updated review. J Anim Behav Biometeorol.

[8] Pritchard LE, White A (2007). Neuropeptide processing and its impact on melanocortin pathways. Endocrinology.

[9] Ducrest A-L, Keller L, Roulin A (2008). Pleiotropy in the melanocortin system, coloration and behavioural syndromes. Trends Ecol Evol.

[10] Shadiack AM, Sharma SD, Earle DC, Spana C, Hallam TJ (2007). Melanocortins in the treatment of male and female sexual dysfunction. Curr Top Med Chem.

[11] Nejad JG, Lee H-G (2023). Coat colour affects cortisol and serotonin levels in the serum and hairs of Holstein dairy cows exposed to cold winter. Domest Anim Endocrinol.

[12] Nejad J, Kim BW, Lee BH, Sung KI (2017). Coat and hair color: hair cortisol and serotonin levels in lactating Holstein cows under heat stress conditions. Anim Sci J.

[13] Morrell J (2020). Heat stress and bull fertility. Theriogenology.

[14] Kendall P, Webster J (2009). Season and physiological status affects the circadian body temperature rhythm of dairy cows. Livest Sci.

[15] Habeeb AA, Gad AE, Atta MA (2018). Temperature-humidity indices as indicators to heat stress of climatic conditions with relation to production and reproduction of farm animals. Int J Biotechnol Recent Adv.

[16] El-Sherbiny HR, Fathi M, Samir H, Abdelnaby EA (2022). Supplemental dietary curcumin improves testicular hemodynamics, testosterone levels, and semen quality in Baladi bucks in the non-breeding season. Theriogenology.

[17] Samir H, Nyametease P, Elbadawy M, Nagaoka K, Sasaki K, Watanabe G (2020). Administration of melatonin improves testicular blood flow, circulating hormones, and semen quality in Shiba goats. Theriogenology.

[18] El-Sherbiny H, Abdelnaby E, Samir H, Fathi M (2022). Addition of autologous platelet rich plasma to semen extender enhances cryotolerance and fertilizing capacity of buffalo bull spermatozoa. Theriogenology.

[19] Fadl AM, Abdelnaby EA, El-Sherbiny HR (2022). INRA82 extender enhances semen quality in ram under cooled and cryopreserved stages. Asian Pac J Reprod.

[20] Fathi M, Salama A, El-Shahat K, EL-Sherbiny HR, Abdelnaby EA (2021). Effect of melatonin supplementation during IVM of dromedary camel oocytes (Camelus dromedarius) on their maturation, fertilization, and developmental rates in vitro. Theriogenology.

[21] El-Seadawy IE, Kotp MS, El-Maaty AMA, Fadl AM, El-Sherbiny HR, Abdelnaby EA (2022). The impact of varying doses of moringa leaf methanolic extract supplementation in the cryopreservation media on sperm quality, oxidants, and antioxidant capacity of frozen-thawed ram sperm. Trop Anim Health Prod.

[22] El-Sherbiny HR, El-Shalofy AS, Samir H (2022). Exogenous L-carnitine administration ameliorates the adverse effects of heat stress on testicular hemodynamics, echotexture, and total antioxidant capacity in rams. Front Vet Sci.

[23] Maurya V, Sejian V, Kumar D, Naqvi S (2010). Effect of induced body condition score differences on sexual behavior, scrotal measurements, semen attributes and endocrine responses in Malpura rams under hot semi-arid environment. J Anim Physiol Anim Nutr.

[24] McManus C, Paludo GR, Louvandini H, Gugel R, Sasaki LCB, Paiva SR (2009). Heat tolerance in brazilian sheep: physiological and blood parameters. Trop Ani Health Prod.

[25] Daramola J, Adeloye A (2009). Physiological adaptation to the humid tropics with special reference to the west african dwarf (WAD) goat. Trop Ani Health Prod.

[26] Gupta M, Mondal T (2021). Heat stress and thermoregulatory responses of goats: a review. Biol Rhyth Res.

[27] Abioja M, Logunleko M, Majekodunmi B, Adekunle E, Shittu O, Odeyemi A, et al. Roles of candidate genes in the adaptation of goats to heat stress: a review. Small Rumin Res. 2022;106878. 10.1016/j.smallrumres.2022.106878.

[28] Joy A, Dunshea FR, Leury BJ, Clarke IJ, DiGiacomo K, Chauhan SS (2020). Resilience of small ruminants to climate change and increased environmental temperature: a review. Animals.

[29] Hashem NM, El-Sherbiny HR, Fathi M, Abdelnaby EA (2022). Nanodelivery System for Ovsynch Protocol improves ovarian response, ovarian blood Flow Doppler Velocities, and Hormonal Profile of Goats. Animals.

[30] Samir H, Nyametease P, Nagaoka K, Watanabe G (2018). Effect of seasonality on testicular blood flow as determined by color Doppler ultrasonography and hormonal profiles in Shiba goats. Anim Reprod Sci.

[31] Hedia MG, El-Belely MS, Ismail ST, Abo El‐Maaty AM (2020). Seasonal variation in testicular blood flow dynamics and their relation to systemic and testicular oxidant/antioxidant biomarkers and androgens in rams. Reprod Domest Anim.

[32] Shahat AM, Thundathil JC, Kastelic JP (2022). Melatonin improves testicular hemodynamics and sperm quality in rams subjected to mild testicular heat stress. Theriogenology.

[33] Samir H, El-Sherbiny H, El-Shalofy A. Seasonal alterations in testicular hemodynamics and echotexture in relation to semen quality in buffalo bulls. Andrologia. 2023; 2023: 5003366. 10.1155/2023/5003366.

[34] Rizzoto G, Ferreira J, Garcia HM, Teixeira-Neto F, Bardella L, Martins C (2020). Short-term testicular warming under anesthesia causes similar increases in testicular blood flow in Bos taurus versus Bos indicus bulls, but no apparent hypoxia. Theriogenology.

[35] Modun D, Giustarini D, Tsikas D. Nitric oxide-related oxidative stress and redox status in health and disease. Oxid Med Cell Longev. 2014; 2014: 129651. 10.1155/2014/129651. 10.1155/2014/129651 PMC4134829 25170388

[36] Walford G, Loscalzo J (2003). Nitric oxide in vascular biology. J Thromb Haemo.

[37] Abdelnaby EA, Yasin NA, Abouelela YS, Rashad E, Daghash SM, El-Sherbiny HR (2022). Ovarian, uterine, and luteal vascular perfusions during follicular and luteal phases in the adult cyclic female rabbits with special orientation to their histological detection of hormone receptor. BMC Vet Res.

[38] Venkatesh K, Mishra C, Pradhan SK, Behera K, Mishra SR, Nayak G (2023). A novel heterozygote allele in caprine melanocortin 1 receptor (MC1R) gene: an association with heat stress traits. Trop Anim Health Prod.

[39] Brito LF, Silva AE, Barbosa RT, Kastelic JP (2004). Testicular thermoregulation in Bos indicus, crossbred and Bos taurus bulls: relationship with scrotal, testicular vascular cone and testicular morphology, and effects on semen quality and sperm production. Theriogenology.

[40] Rizzoto G, Ferreira J, Codognoto V, Oliveira K, García HM, Pupulim A (2020). Testicular hyperthermia reduces testosterone concentrations and alters gene expression in testes of Nelore bulls. Theriogenology.

[41] Hedia M, El-Belely M, Ismail S, El-Maaty AMA (2019). Monthly changes in testicular blood flow dynamics and their association with testicular volume, plasma steroid hormones profile and semen characteristics in rams. Theriogenology.

[42] Lin P-H, Huang K-H, Tian Y-F, Lin C-H, Chao C-M, Tang L-Y (2021). Exertional heat stroke on fertility, erectile function, and testicular morphology in male rats. Sci Rep.

[43] Thanh TN, Van PD, Cong TD, Le Minh T, Vu QHN (2020). Assessment of testis histopathological changes and spermatogenesis in male mice exposed to chronic scrotal heat stress. J Anim Behav Biometeorol.

[44] Camela ES, Nociti RP, Santos VJ, Macente BI, Murawski M, Vicente WR (2019). Changes in testicular size, echotexture, and arterial blood flow associated with the attainment of puberty in Dorper rams raised in a subtropical climate. Reprod Domest Anim.

[45] Acharya R, Gupta U, Sehgal J, Singh M (1995). Coat characteristics of goats in relation to heat tolerance in the hot tropics. Small Rumin Res.

[46] El-Sherbiny H, Shahat A, Hedia M, El-Shalofy A (2022). Effect of sexual maturation on testicular morphometry and echotexture and their association with intratesticular blood flow in ossimi rams. Indian J Small Rumin.

[47] Aleena J, Sejian V, Bagath M, Krishnan G, Beena V, Bhatta R (2018). Resilience of three indigenous goat breeds to heat stress based on phenotypic traits and PBMC HSP70 expression. Int J Biometeorol.

[48] Sezen O, GÜNEY O (2010). Physiological responses and some blood parameters of bucks under Mediterranean climate conditions. Anadolu Tarım Bilimleri Dergisi.

[49] Odubote I (1994). Characterization of the west african dwarf goat for certain qualitative traits. Nigerian J Anim Prod.

[50] Rout P, Kaushik R, Ramachandran N (2016). Differential expression pattern of heat shock protein 70 gene in tissues and heat stress phenotypes in goats during peak heat stress period. Cell Stress chaperones.

